# Character-based DNA barcoding for authentication and conservation of IUCN Red listed threatened species of genus *Decalepis* (Apocynaceae)

**DOI:** 10.1038/s41598-017-14887-8

**Published:** 2017-11-02

**Authors:** Priyanka Mishra, Amit Kumar, Gokul Sivaraman, Ashutosh K. Shukla, Ravikumar Kaliamoorthy, Adrian Slater, Sundaresan Velusamy

**Affiliations:** 1 Plant Biology and Systematics, CSIR - Central Institute of Medicinal and Aromatic Plants, Research Center, Allalsandra, GKVK Post, Bengaluru, 560065 Karnataka India; 2 Biotechnology Division, CSIR - Central Institute of Medicinal and Aromatic Plants, P.O. CIMAP, Lucknow, 226015 Uttar Pradesh India; 3 School of Conservation, TransDisciplinary University, 74/2, Jarakabande Kaval, Post Attur, Via Yelahanka, Bangalore, 560064 Karnataka India; 4 Biomolecular Technology Group, Faculty of Health and Life Sciences, De Montfort University, Leicester, LE1 9BH UK

**Keywords:** Plant evolution, Plant molecular biology

## Abstract

The steno-endemic species of genus *Decalepis* are highly threatened by destructive wild harvesting. The medicinally important fleshy tuberous roots of *Decalepis hamiltonii* are traded as substitute, to meet the international market demand of *Hemidesmus indicus*. In addition, the tuberous roots of all three species of *Decalepis* possess similar exudates and texture, which challenges the ability of conventional techniques alone to perform accurate species authentication. This study was undertaken to generate DNA barcodes that could be utilized in monitoring and curtailing the illegal trade of these endangered species. The DNA barcode reference library was developed in BOLD database platform for candidate barcodes *rbcL*, *matK*, *psbA-trnH*, *ITS* and *ITS2*. The average intra-specific variations (0–0.27%) were less than the distance to nearest neighbour (0.4–11.67%) with *matK* and *ITS*. Anchoring the coding region *rbcL* in multigene tiered approach, the combination *rbcL* + *matK* + *ITS* yielded 100% species resolution, using the least number of loci combinations either with PAUP or BLOG methods to support a character-based approach. Species-specific SNP position (230 bp) in the *matK* region that is characteristic of *D*. *hamiltonii* could be used to design specific assays, enhancing its applicability for direct use in CITES enforcement for distinguishing it from *H*. *indicus*.

## Introduction

**Figure 1:**
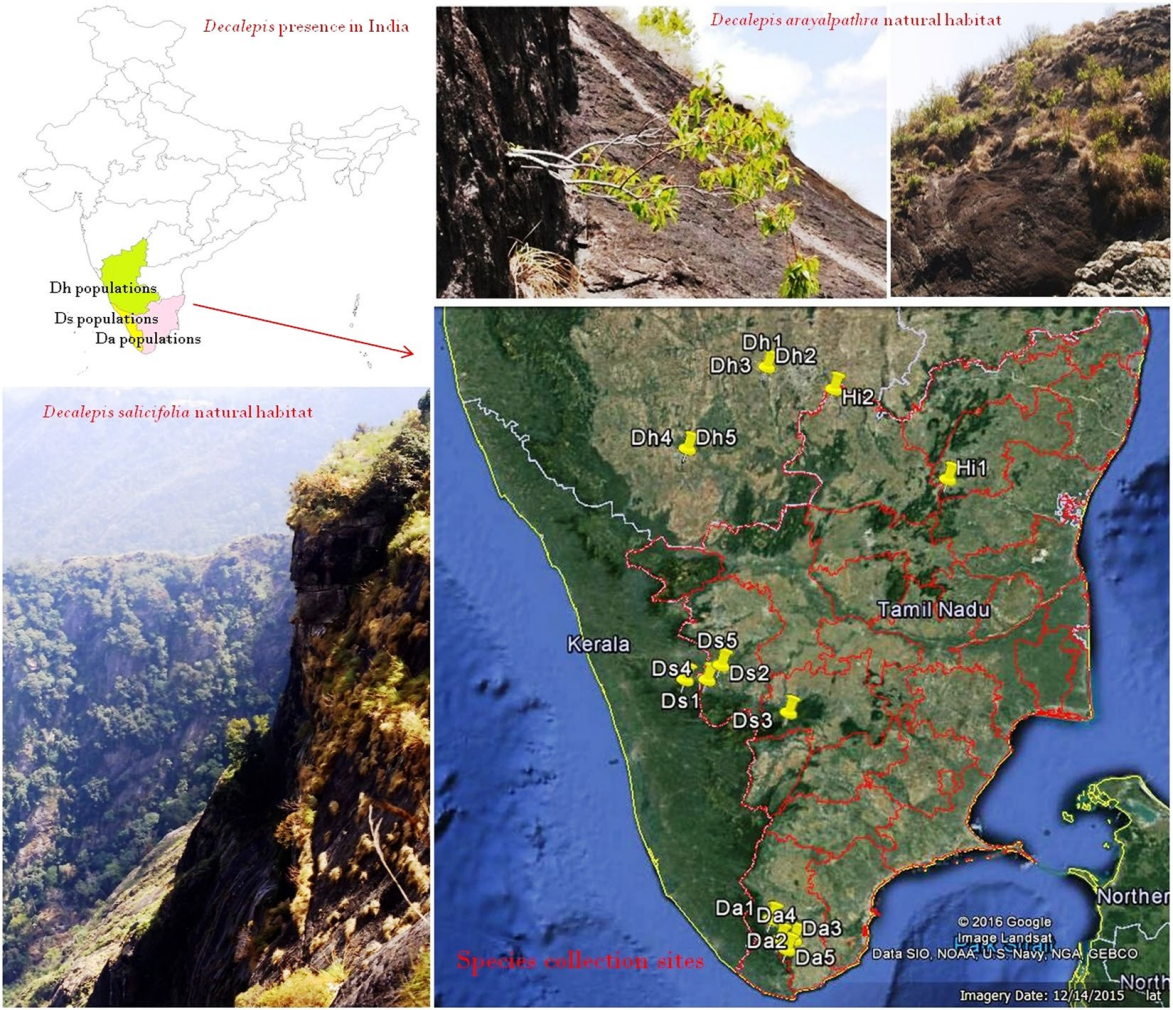
Geographical mapping of *Decalepis* species in India. Maps were generated through tools in Google Earth program version 7.1.7.2606 (https://www.neowin.net/news/google-earth-pro-7172606) based on the recorded GPS (Garmin) coordinates on the collection sites.

*Decalepis arayalpathra* (J. Joseph & V. Chandras.) Venter (Apocynaceae), locally known as Amirthapala, is a steno-endemic species in Eastern and Western ghats of peninsular India, one of the world’s eight hottest hotspots of biodiversity^1,2^. Previously recognized as *Janakia arayalpathra* (J. Joseph & V. Chandras.) Venter, the synonym *D. arayalpathra* has now been placed as a monotypic genus along with *Utleria salicifolia* and *Decalepis hamiltonii* in the family Periplocaceae Schltr. Taxonomic revisions at the family level recorded *D. arayalpathra* (J. Joseph & V. Chandras.) Venter, *D. hamiltonii* (Wight & Arn.), and *D. salicifolia* (Bedd. ex Hook.f.) Venter as the accepted names for the species under the family Apocynaceae^3,4^, http://www.theplantlist.org. The species are highly endemic and are restricted to dry and moist deciduous forest segments of Tamil Nadu, Kerala and Andhra Pradesh in India (Fig. 1). The plants are distributed in grassy patches of exposed rocky slopes, which grow in clumps without any firm holding (Fig. 1) and are exposed to heavy wind velocity, high temperature and moderately good rainfall throughout most of the year^5^. Species of *Decalepis* were assessed as a Critically Endangered—Globally (CR-G), Red listed medicinal plant species by the International Union for Conservation of Nature (IUCN) and declared as a species with high conservation concern by the National Biodiversity Authority of India (NBA)^6–8^, http://www.iucnredlist.org/details/50126587/0. The plants are utilized in traditional Indian and Chinese medicine for treatments of disorders related to the digestive system, lungs and circulatory system. The highly aromatic tuberous roots are used for peptic ulcer, stomach-ache, cancer-like afflictions, etc. and as a rejuvenating tonic by the native Kani tribe of Southern Western Ghats with significant gastric antisecretory and antiulcer activities^2,9,10^. Phytochemically, the presence of 2-hydroxy-4-methoxybenzaldehyde (98%) in the tuberous root oil of *D. arayalpathra* renders it as a valuable source for the production of commercially important flavour compound vanillin^11^. The presence of similar aroma reported in the roots of *D. hamiltonii* and *D. salicifolia* along with their exudation and texture (Fig. S1a–f) place them as a potential substitute in markets for *D. arayalpathra*.

*H. indicus* is a well-known drug in Ayurvedic and Unani systems of medicine. The plant possesses potential anti-cancerous, immunomodulatory, anti-ulcer, antioxidant, hepatoprotective, anti-inflammatory, antihyperglycemic, anti-diarrhoeal, anti-venom and antimicrobial properties^12,13^. The commercially important plant part used therapeutically is the root, which finds extensive use as a blood purifier, tonic, diuretic and diaphoretic by the tribals of India^14^. The roots of *H. indicus* are very thin and short, and are firmly attached to the soil (Fig. S1h), which requires extensive labour and time to dig out from the ground while those of *D. hamiltonii* are large, fleshy and loosely attached to the soil (Fig. S1d–f) and are therefore widely used as a substitute by the Indian drug industries for the preparation of the Ayurvedic formulation *Sariba* (Indian Sarsaparilla), whose botanical identity is *H. indicus*. In order to meet the high global market demand of *Hemidesmus* roots, the healthy and fleshy tuberous roots of *D. hamiltonii* are traded as a substitute in thousands of tonnes every year from the uncultivated wild sources^7,15^. The indiscriminate collection from the wild has put tremendous pressure on the species survival. Thus, proper identification and authentication of these plants are needed for their sustained use, especially for the threatened species, whose trade is regulated by Convention on International Trade of Endangered Species (CITES).

In our recent study to evaluate genetic diversity and population structure in the wild populations of *D. arayalpathra* based on demographic study and genetic data realized through marker assays, we demonstrated the occurrence of low genetic diversity and high genetic differentiation between the populations^16^. In addition, the populations were recorded to have restricted distribution and high fragmentation and were found to be over-exploited by destructive harvesting. Niche specificity, damage by fruit wasp, population bottleneck, restricted gene flow, and root rot by fungus are the various factors, which have endangered this group in their wild habitat^16,17^. This signals the need for taxon recognition in biodiversity hotspots, which is a key factor for the enforcement of plant protection regulations and the future conservation of the species^18^.

The conservation and management of critically endangered and threatened species primarily focus on the correct identification and delimitation of the target species, thereby improving the status of global biodiversity through the Convention on Biological Diversity 2020^19^. Conventional methods to identify raw drugs and plant material at the species level are not always feasible due to limitations inherent in morphology-based systems and the dwindling pool of taxonomists^20^. For the last decade, microgenomic identification systems have provided a promising approach towards the diagnosis of biological diversity^21^, with DNA barcoding becoming popular. The diversity among DNA sequences used to identify taxa can be viewed as genetic barcodes^22^. The haploid, uniparentally-inherited mitochondrial region-based single locus DNA barcode *COI*, in combination with well-developed universal primer sets, resulted in the routine recovery of high-quality sequences from animal clades^23^. Translating these principles into the selection of suitable barcoding region in plants has proved elusive. The plant mitochondrial genome has certain constraints, which precludes its use as a universal plant barcode^24^. The quest shifted towards the plastid and nuclear-based regions, following initial *in silico* and laboratory-based evaluations of different coding and non-coding markers. The outcome of these trials proposed major individual candidate regions *matK*, *rbcL*, *rpoB, rpoC1*, and the intergenic spacers *ITS*, *psbA-trnH*, *trnL-F*, *atpF-atpH* and *psbK-psbI*, etc. for use in plants based on their discrimination capacity^25,26^. Due to pitfalls and challenges associated with a single locus, the combination of loci emerged as a promising choice to obtain appropriate species discrimination^27–29^. The Consortium for the Barcode of Life (CBOL), proposed *rbcL* + *matK* as a standard two-locus barcode for all land plants, but based on further refinement, suggested the need for the addition of supplementary loci, viz. the non-coding cpDNA *psbA-trnH* intergenic spacer and nuclear ribosomal internal transcribed spacer (*nrITS* & *nrITS2*) regions^30–33^.

Hitherto, many researchers have evaluated the combination of several proposed plastid and nuclear regions to envisage the universal barcode in plants through their comprehensive studies in taxonomically complex groups^24,25,27,29,34–36^. Currently, the barcoding research is shifting beyond this evaluation phase. Apart from its practical application to provide insights into species-level taxonomy, the technology is being acknowledged as an effective tool by providing pretentious discriminatory power for species in trade (CITES listed), forensic identification, and ecological forensics as well as species identification for rare, threatened and endangered plant groups^37–39^. The potentiality of DNA barcodes to identify the species even from a minute amount of tissue (rather than a whole plant, preferably in flowering stage, as required in the current taxonomic methods) is augmenting the taxonomic tool box by tackling illegal trade of endangered species.

In the absence of a single consensus universal plant barcode, it becomes obligatory to determine the optimal region(s) according to the taxa of interest. The search for a suitable barcode for the genus *Decalepis* is completely lacking. Therefore, this study was designed to establish the first ever reference library, using the most effective barcode(s) to provide molecular identity to the threatened and endemic species of *Decalepis*. The efficacy of different analytical approaches of DNA barcoding data will be evaluated to test the discrimination ability of the chosen markers for *Decalepis*. The findings from this study, in corroboration with the population dynamics proposed in our recently published research^16^, will provide the valuable tools needed to develop a standard protocol to catalogue species identity in CITES enforcement, and to develop conservation plans for the management of threatened species of this group.

## Results

### PCR amplification and sequencing success rate

A total of 17 individuals representing all the three species of the genus *Decalepis* and the species *H. indicus*, were successfully amplified and sequenced using five DNA barcodes *rbcL*, *matK*, *psbA-trnH*, *ITS* and *ITS2*. The PCR and sequencing success rate for each of the five regions was 100% with regards to the universality of primers (Table 1). The newly generated 85 sequences were submitted to GenBank (Table 2). Since no barcoding studies have been done to date for the genus *Decalepis*, we found only two congeneric sequences with accession number KP764847.1 and DQ916845.1 corresponding to *D. salicifolia* and *D. arayalpathra* in NCBI BLAST hits. Thus, the database sequences were not included in our analysis. Among the obtained hits, the sequences showed most similarity with other genera of the family Apocynaceae. The PCR amplicons of all the five loci showed a size range consistent with the mean size of the respective marker (Table S1). The sequence characteristics of all the studied barcodes have been tabulated in Table 1. *ITS* sequences ranged from 663 bp to 666 bp with 94 variable sites and 80 informative sites. The alignment length was 679 bp with 19 indels of 1–3 bp within the aligned region. The primers for *ITS* used in the study lie in the conserved flanking regions of 18S and 26S, so the sequences were trimmed to the regions of *ITS1, 5.8S* and *ITS2*. The *ITS2* region also showed 6 indels of 1–2 bp within the aligned region of 406 bp. The numbers of variable sites were 55 with 54 informative sites. All the three plastid genes *rbcL, matK*, and *psbA-trnH* were without indels, with aligned lengths of 676 bp, 751 bp and 380 bp, respectively. The coding region *rbcL* was found to be highly conserved (from 416 bp to 642 bp) within the species of *Decalepis*, resulting in 664 conserved sites among the 676 bp aligned region.

**Table 1:** Sequence characteristics of the five DNA barcode loci evaluated in this study.

Parameters assessed	DNA barcode locus				
	*rbcL*	*matK*	*psbA-trnH*	*ITS*	*ITS2*
Number of individuals	17	17	17	17	17
PCR Success (%)	100	100	100	100	100
Sequencing success (%)	100	100	100	100	100
Sequence length (bp)	676	751	376	663–666	400
Aligned length (bp)	676	751	380	679	406
No. of variable sites	12	15	29	94	55
No. of indels	0	0	0	19	6
No. of Parsimony informative sites	11	12	13	80	54
Pairwise identity (%)	99.4	99.3	98.2	95.8	95.5

**Table 2:** Taxon sampling, BOLD database details and GenBank accession numbers of the reference library for *Decalepis* species generated in this study.

Species (Number of individuals)	Sampling locations	BOLD database sample Id	GenBank accession numbers				
			*rbcL*	*matK*	*trnH-psbA*	*ITS*	*ITS2*
*Decalepis arayalpathra* (5)	Kuthuraikattimottai; Tirunelveli	CRCBDa1	KX528330	KT273997	KT362274	KT338784	KT362291
	Nadukandanparai; Tirunelveli	CRCBDa2	KX528333	KT273998	KT362275	KT338785	KT362292
	Nambikoil, KMTR; Tirunelveli	CRCBDa3	KX377975	KT273999	KT362276	KT338786	KT362293
	Maramalai; Kanyakumari	CRCBDa4	KX528332	KT274000	KT362277	KT338787	KT362294
	Asambu; Kanyakumari	CRCBDa5	KX528331	KT274001	KT362278	KT338788	KT362295
*Decalepis hamiltonii* (5)	Savandurga; Bangalore Rural	CRCBDh1	KX618637	KT279711	KT362279	KT338789	KT362296
	Savandurga; Bangalore Rural	CRCBDh2	KX643354	KT279712	KT362280	KT338790	KT362297
	Savandurga; Bangalore Rural	CRCBDh3	KX643355	KT279713	KT362281	KT338791	KT362298
	Chamundi hills; Mysore	CRCBDh4	KX643356	KT279714	KT362282	KY072831	KT362299
	Chamundi hills; Mysore	CRCBDh5	KX809592	KT279715	KT362283	KT338792	KT362300
*Decalepis salicifolia* (5)	Topslip; Coimbatore	CRCBDs1	KX668215	KT279722	KT362284	KT338793	KT362301
	Kathadimudi Peak; Coimbatore	CRCBDs2	KX668216	KT279723	KT362285	KT338799	KT362302
	Vattakandal Shola; Kodaikanal	CRCBDs3	KX668217	KT279724	KT362286	KT338798	KY072830
	Topslip; Coimbatore	CRCBDs4	KX668218	KT279725	KT362287	KT338794	KT362303
	Kathadimudi Peak; Coimbatore	CRCBDs5	KX809593	KT279726	KT362288	KT338795	KT362304
*Hemidesmus indicus* (2)	Valasamalai; Tiruvannamalai	CRCBHi1	KX711546	KT279727	KT362289	KT338796	KT362305
	Hosur; Krishnagiri	CRCBHi2	KX711547	KT279728	KT362290	KT338797	KT362306

### Distance analysis and barcoding regions for species identification

**Figure 2:**
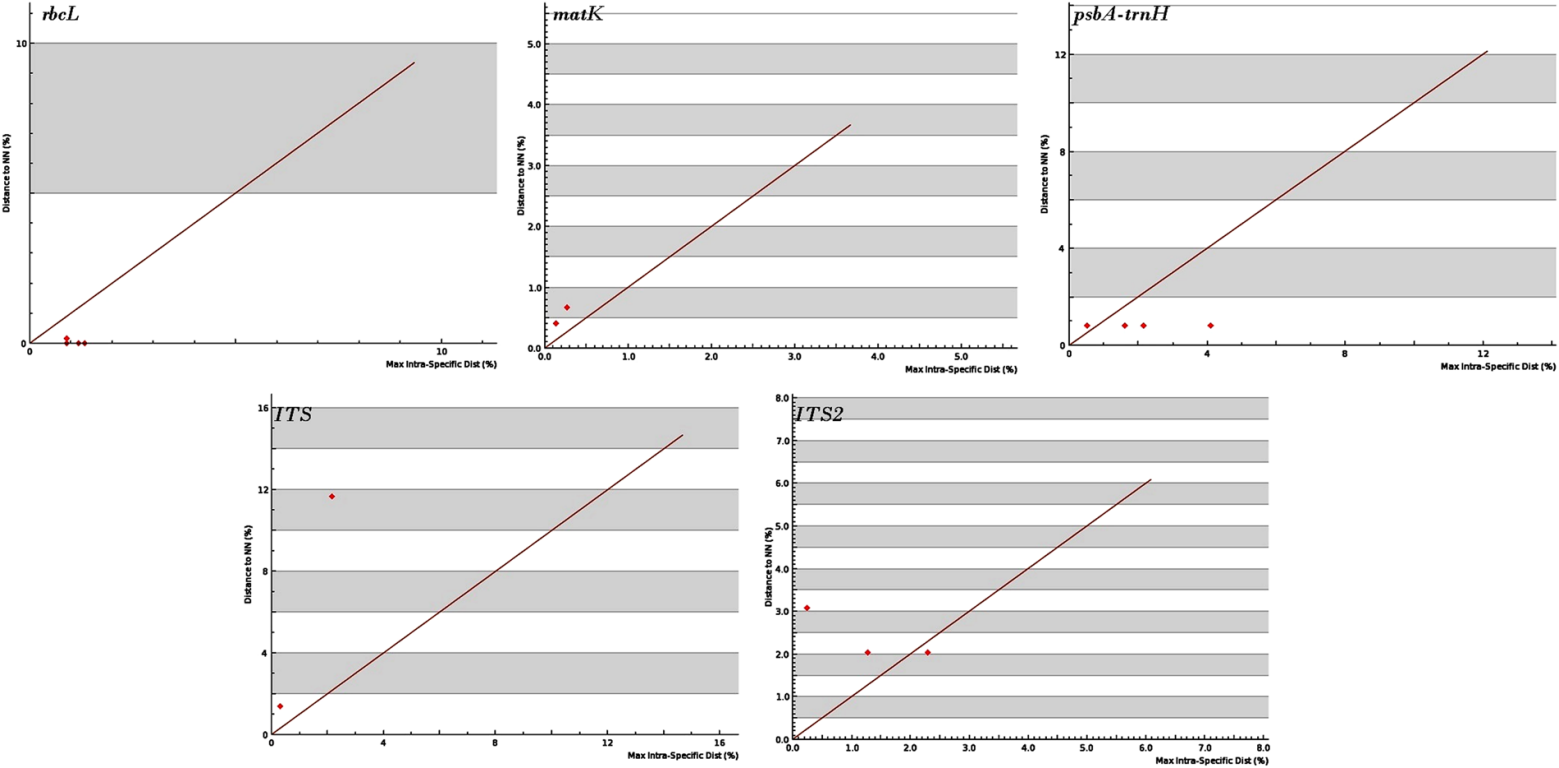
Barcode gap plot for the five individual barcodes. The distances to the nearest neighbor (NN) vs. the maximum intra-specific distances (%) realized through Kimura-2-parameter (K2P) were plotted for species discrimination. Each dot represents one or several individuals since they share identical values of intra-specific and inter-specific distances. Dots above the 1:1 line indicated the presence of a barcode gap.

Barcode gap analysis provides the distribution of distances within conspecifics and the distance to the nearest neighbour (NN) of each species. The analysis of the distance to the nearest non-conspecific against the distance to the furthest conspecific among the corresponding sequences, based on the K2P genetic distance method, revealed that the mean intra-specific distances were less than the distance to the nearest neighbour, in the cases of *matK, ITS* and *ITS2* loci (Table S2). These three candidate barcodes demonstrated the existence of a clear barcode gap, which is ideal for species identification (Fig. 2). On the other hand, *rbcL* and *psbA-trnH* exhibited maximum intra-specific divergence of 1.34% and 4.11%, respectively among the individuals of *D*. *salicifolia*. Among the individuals of *D*. *arayalpathra*, the highest distance of 1.62% was recorded with *psbA-trnH*. *D. hamiltonii*, *H. indicus*, and *D. salicifolia* shared the maximum identity in terms of their nearest neighbour. *D. hamiltonii* and *H. indicus* recorded the lowest NN distance of 0.4% (*matK*) amongst the four species, which makes *D. hamiltonii* a potent substitute for *H. indicus* in the market. The nuclear region *ITS* and *ITS*2 showed maximum inter-specific distances (1.37% to 11.67%) among all the 4 species, revealing them to be potent loci, along with *matK*, to discriminate the species of genus *Decalepis*. On the other hand, the coding region *rbcL* showed only 0–0.15% divergence with any of the nearest neighbours among *D. arayalpathra*, *D. hamiltonii*, *D. salicifolia* and *H. indicus* (Table S2). Figure 2 depicts the scatter plot of the maximum intra-specific distances against the NN distances to confirm the existence and magnitude of the barcode gap with all the five candidate barcodes. Maximum intra-specific distances were less than 2% in all species except *D. salicifolia* (4.11%) and *H. indicus* (2.15%).

**Figure 3:**
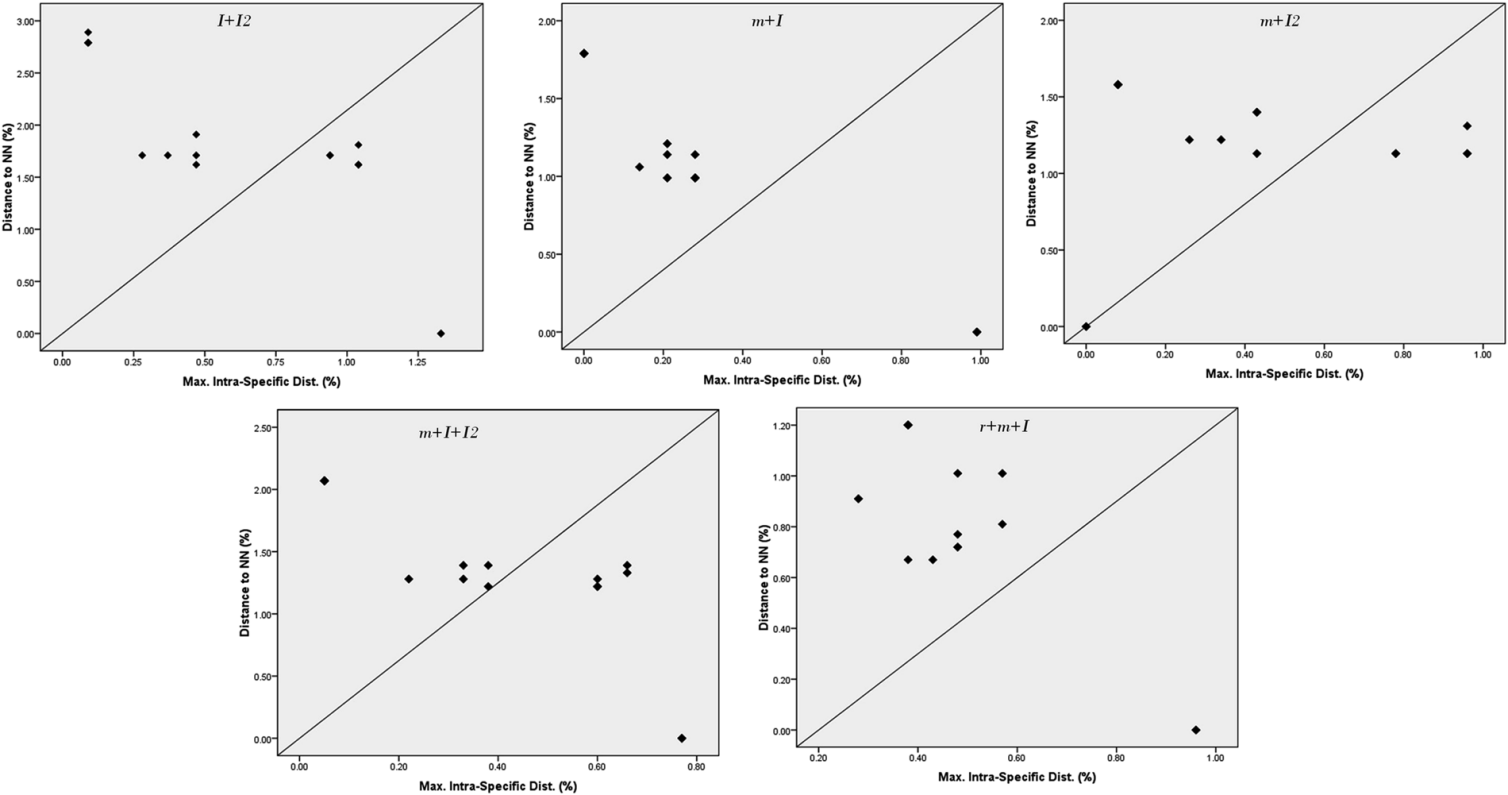
Evaluation of barcode gaps for the favourable barcode combinations in *Decalepis* species. Distances to the nearest neighbor (NN) were plotted against maximum intra-specific Kimura-2-parameter (K2P) distances (%). Each dot represents one or several individuals since they share identical values of intra-specific and inter-specific distances. Dots above the 1:1 line indicated the presence of a barcode gap.

Based on the utility of individual loci, *matK, ITS* and *ITS2* were the favourable choices in the genus *Decalepis* and the regions were combined with other loci to assess their resolution rate. Table S3 presents the details of the obtained range of inter and intra-specific distances with all the possible combinations of various loci. From among the 26 combinations undertaken in the study, most of them showed the clear presence of a barcoding gap, which reflects the adequacy of the multilocus approach in plant DNA barcoding. The core barcode *rbcL* + *matK* showed a slight overlap of between 0.0–0.1% among the individuals of the species with their nearest neighbour (Fig. S2). However, complementing the barcode with the non-coding locus *ITS* at the third position improved the resolution with its nearest neighbour, with a clear barcoding gap (Figs 3 and S2). All the possible combinations of *matK*, *ITS* and *ITS2* showed no intra-specific divergence among the individuals based on pairwise genetic distances and their frequency distribution (Fig. S2). The lowest average intra-specific distance [0.000 ( ± 0.000) – 0.013 ( ± 0.003) %] and highest average inter-specific distance [0.019 ( ± 0.004) – 0.128 ( ± 0.011) %] was observed with the combination of *ITS* + *ITS2*. Complementing them with the plastid locus *matK* resulted in a range of 0.000 (±0.000)–0.007 (±0.001) % intra-specific distances and 0.014 (±0.003)–0.074 (±0.007) % NN distance (Table S3). The combinations of *matK* + *ITS* + *ITS2* and *rbcL* + *matK* + *ITS* loci shared 97.2% and 98.2% pairwise identity among their residues. Based on the observations of genetic distances among the species, the core barcode *rbcL* + *matK* along with the addition of *ITS* at third position, and the combination of *matK* + *ITS* + *ITS2* would both be favourable choices for barcoding the endangered plant species of *Decalepis*. Though most of the other loci combinations resulted in the presence of a significant barcoding gap with high genetic distances with their nearest neighbour, they also recorded an intra-specific bias among the individuals.

### Phylogenetic analysis of *Decalepis* species based on parsimony method

For estimating the evolutionary divergences among the species of genus *Decalepis*, we employed distance-based (NJ) and character-based (MP) methods on all the barcode regions. The results of the criterion-based approach outperformed the distance-based NJ method in assigning individual characters to the tree. Since the characters are reduced to distances in the NJ methods, which sometimes get lost in the pairwise comparisons and result in biased distances, further analyses were carried out using the MP model in PAUP.

**Figure 4:**
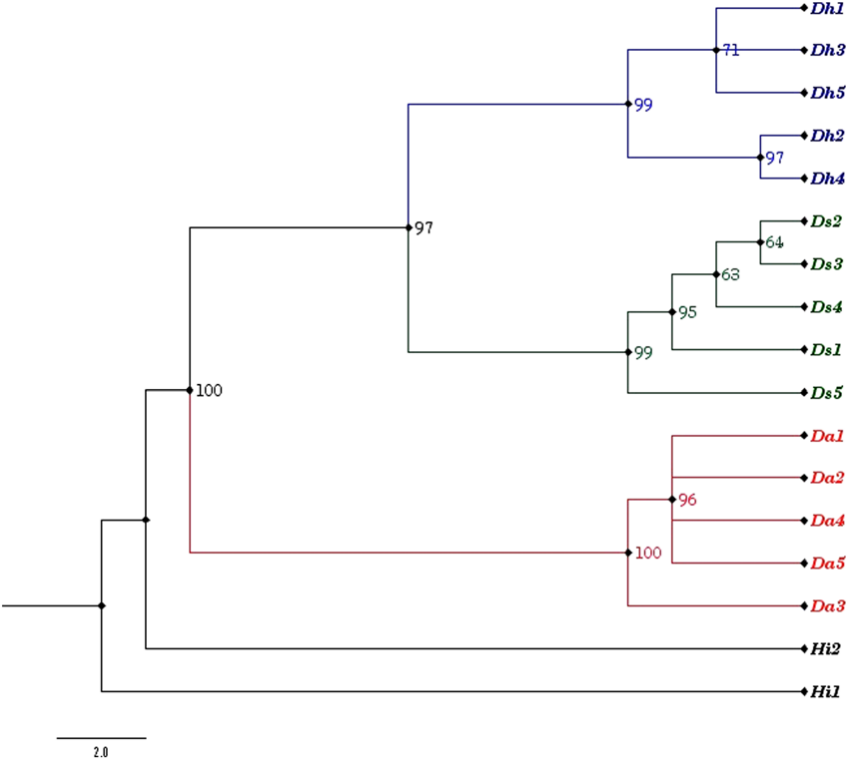
Strict consensus tree showing the relationship of *Decalepis* species resulting from maximum parsimony analysis using the barcode *rbcL* + *matK* + *ITS*. Tree length = 146, CI = 85%, RI = 91, RC = 78%. Bootstrap support values below 60% are not shown. Individuals corresponding to species monophyly: Red: *D. arayalpathra*, Green: *D. salicifolia*, Blue: *D. hamiltonii*.

The evaluation of each barcode locus and combination of loci based on computational phylogenetics, showed similar tree topologies, in agreement with the barcoding gap analysis. The heuristic search of the set of taxa presented a reticulated hypothesis based on the underlying algorithms with reliable clade support. The most favourable barcode dataset *rbcL* + *matK* + *ITS* and *matK* + *ITS* + *ITS2* showed a consistency index (CI) of 85% (CI = 0.8562) and 90% (CI = 0.9024) respectively, with the cladogram. The former dataset for parsimony analysis included 2106 characters, of which 103 were parsimony informative and 18 variable characters were found to be parsimony-uninformative, while the latter dataset contributed 146 informative characters from among 1836 total characters. Thus, the combination of coding and non-coding regions, *rbcL*, *matK* and *ITS*, were concluded to be the best choice for species resolution in genus *Decalepis* (Fig. 4). The strict consensus tree of *rbcL* + *matK* + *ITS* resulted in a tree length of 146 steps with the node supported clade framing the well resolved species of *Decalepis*. The retention index (RI) was 0.9121 and the re-scaled consistency index (RC) was 0.7809. The individuals belonging to *D*. *arayalpathra* showed several polytomies, which makes the intra-specific relationship difficult. However, they formed a clade, which was 100% supported, with *D. hamiltonii*, *D. salicifolia* as sister species. At the large polytomy, *D. hamiltonii* and *D. salicifolia* framed two well supported groups with a 97% bootstrap value. The resulting tree maintained the species monophyly in terms of the *Decalepis* species, and both individuals of *H. indicus* framed the nodal cluster at the base of the tree (Fig. 4).

### Comparison of discrimination methods and barcode regions

The discrimination ability of the all the five candidate barcodes and their 26 possible combinations were compared using TaxonDNA and BLOG. All the barcoding datasets represented equal number of individuals corresponding to respective species. The rates of “correctly identified”, “misidentified” and “not identified” individuals for each dataset and method are shown in Table 3. Averaged over both methods, *matK* (100%), *ITS* (75–100%) and *ITS2* (100%) turned out to have the highest success rate among the single locus barcode. The combinations of loci that included all three of these regions provided higher success rates than other combination barcodes. The best two locus combinations ranged in the order *matK* + *ITS2* (100/100/88.23%) > *matK* + *ITS* (100 + 88.23 + 88.23%) > *ITS* + *ITS2* (100 + 88.23 + 88.23%), based on the BM, BCM and All Species Barcode modules of TaxonDNA. When we compared these loci based on a character-based approach, the combinations proved to be highly successful, reaching 75–100% correct identification for the species using BLOG (Table 3). The *rbcL* region showed very poor discrimination success for single and two-locus barcodes. However, supplementing it with the other loci, with *rbcL* being at the first position, afforded 100% correct identification (100/0/0), using either the TaxonDNA or BLOG based methods. Thus, a tiered approach to barcoding promised the potential to barcode the endangered species of genus *Decalepis*.

**Table 3:** Species identification rates in % (correctly identified/misidentified/not identified) using two different classification methods for each of the five barcodes and their combinations.

Barcode locus/loci	TaxonDNA		BLOG
	Best match (%)	Best close match (%)	
*rbcL*	18/23/59	18/23/58	0/25/75
*matK*	100/0/0	88/0/12	100/0/0
*psbA-trnH*	94/6/0	94/6/0	100/0/0
*ITS*	100/0/0	88/0/12	75/25/0
*ITS2*	100/0/0	100/0/0	100/0/0
*rbcL* + *matK*	82/12/6	83/12/6	100/0/0
*rbcL* + *psbA-trnH*	76/24/0	76/24/0	100/0/0
*rbcL* + *ITS*	100/0/0	88/0/12	75/25/0
*rbcL* + *ITS2*	100/0/0	100/0/0	75/0/25
*matK* + *psbA-trnH*	100/0/0	76/24/0	75/25/0
*matK* + *ITS*	100/0/0	88/0/12	100/0/0
*matK* + *ITS2*	100/0/0	100/0/0	75/0/25
*psbA-trnH* + *ITS*	82/12/6	83/12/6	75/0/25
*psbA-trnH* + *ITS2*	76/24/0	76/24/0	75/0/25
*ITS* + *ITS2*	100/0/0	88/0/12	75/25/0
*rbcL* + *matK* + *psbA-trnH*	100/0/0	88/0/12	100/0/0
*rbcL* + *matK* + *ITS*	100/0/0	100/0/0	100/0/0
*rbcL* + *matK* + *ITS2*	100/0/0	100/0/0	75/0/25
*rbcL* + *psbA-trnH* + *ITS*	100/0/0	88/0/12	100/0/0
*rbcL* + *psbA-trnH* + *ITS2*	100/0/0	100/0/0	75/0/25
*rbcL* + *ITS* + *ITS2*	100/0/0	88/0/12	75/25/0
*matK* + *psbA-trnH* + *ITS*	100/0/0	100/0/0	100/0/0
*matK* + *psbA-trnH* + *ITS2*	100/0/0	100/0/0	75/0/25
*matK* + *ITS* + *ITS2*	100/0/0	88/0/12	100/0/0
*psbA-trnH* + *ITS* + *ITS2*	100/0/0	100/0/0	100/0/0
*rbcL* + *matK* + *psbA-trnH* + *ITS*	100/0/0	88/0/12	100/0/0
*rbcL* + *matK* + *psbA-trnH* + *ITS2*	100/0/0	100/0/0	75/0/25
*rbcL* + *psbA-trnH* + *ITS* + *ITS2*	100/0/0	100/0/0	100/0/0
*rbcL* + *matK* + *ITS* + *ITS2*	100/0/0	100/0/0	75/0/25
*matK* + *psbA-trnH* + *ITS* + *ITS2*	100/0/0	100/0/0	100/0/0
*rbcL* + *matK* + *psbA-trnH* + *ITS* + *ITS2*	100/0/0	100/0/0	100/0/0

TaxonDNA: Best match and Best close match results. Not identified rates are summed over the “Ambiguous” and “No match” categories. Please see supplementary Table S4 for details.

BLOG: percentage correct classification for test file, using 90% slicing at species level (Refer to materials and methods for detailed analysis).

The highest success rate for preferred barcoding options in *Decalepis* are highlighted in grey.

Based on the comparison of methods, both the TaxonDNA and BLOG approaches performed equally well on average across all the favourable barcodes (both provided 75–100% correct identification). However, the rate of misidentification for all the loci was 0% in TaxonDNA but 25% in BLOG (Table 3, highlighted in grey). In contrast, BLOG outperformed TaxonDNA by resulting 0% individuals as “not identified”, whereas ~11.76% of individuals were not identified in TaxonDNA. Phylogenetic analysis of *Decalepis* species based on a character-based approach also resulted in assigning individual characters to the tree. Thus, character-based rather than distance-based methods are the appropriate choice to test the hypothesis.

## Discussion

### The efficiency of barcoding regions in elucidating the molecular identity of *Decalepis* species

The present study is the first published attempt to describe the molecular phylogeny of the threatened and endangered species of *Decalepis*. It shows that barcoding markers can accurately distinguish between the species, revealing homogeneous clades with high resolution assignment of individuals at the species level (Fig. 4). From among the tested plastid and nuclear loci, *ITS* had the highest efficiency as a single locus in the identification of species in *Decalepis* (Fig. S3). The high copy number of *rRNA* genes, greater discriminatory power at low taxonomic levels and higher evolutionary rate makes *ITS* a promising locus in plant molecular systematics^40^. The better phylogenetic signalling of *ITS* compared to the plastid barcoding markers in *Decalepis* is compatible with the results of other genus-level studies in *Passiflora*^41^, *Euphorbia*^42^, *Paeonia*^43^ and *Melilotus*^44^, amongst others.

The two barcodes *rbcL* and *psbA-trnH* had the lowest discriminatory power as a single locus, which limits their utility in *Decalepis*, despite their value for barcoding of other plant groups^20,24^. Both regions failed to discriminate between the species, and the resulting phylogenetic tree showed huge over-mixing of individuals with poor clade support. The potential substitute, *H. indicus*, grouped with *D. hamiltonii*, and the individuals of *D. salicifolia* were found to be unresolved at the base of the tree (Fig. S3). The problems of alignment ambiguities and frequent inversions associated with palindromic sequences within the *psbA-trnH* region have been found in multiple lineages of Angiosperms and possibly complicate its use as a barcode, especially if they occur within species^29^. The suitability of the chloroplast region *rbcL* for studies of molecular evolution at the species level has been controversial, in part due to its ~1430 bp length. For clear species discrimination, the entire region needs to be sequenced, which limits its use as a barcoding sequence. The ideal barcoding region should be short enough to amplify, and amenable to analysis through single-pass sequencing^35^. However, complementing the region with other barcoding marker(s) improves its discrimination ability, as shown in earlier studies^29,31,35,45^.

The chloroplast *matK* coding region presented a better credentials as a candidate barcode, showing both high sequence recovery and high identification rates either as a single locus or in combination with *ITS*. The *matK* + *ITS* combination framed the entire sister species of *Decalepis* as a major cluster, with *H. indicus* positioned as an out-group at a nodal branch at the base of the tree (Fig. 4). The chloroplast gene *matK* showed a higher rate of nucleotide substitutions than other tested loci from the plastid genome, which provided higher inter-specific divergence values among *matK* sequences. The nuclear two-locus barcode combination of *ITS* + *ITS2* also showed a closely similar result, which confirms the advantage of the multi-locus consensus barcode approach in plant DNA barcoding.

In the effort to develop molecular identification methods for the species of *Decalepis* for the purpose of CITES control, the focus is to provide a clear resolution of sister species and of potential substitutes. The search for a universal DNA barcode for plants led to the recommendation by CBOL of the combination two-locus barcode *rbcL* + *matK*^29^. In this study, we have performed a comprehensive evaluation of all the 26 possible (single, two- and three-locus) combinations of the two recommended barcodes plus three supplementary candidate regions. Amongst all the three locus barcode combinations investigated here, *rbcL* + *matK* + *ITS* provided the best identification in maintaining the species monophyly in *Decalepis* (Fig. 4). Most importantly, the closely related species that were prone to substitution or adulteration, such as *D. hamiltonii* and *H. indicus*, could be accurately identified by the combination barcode *rbcL* + *matK* + *ITS*. In particular, the rapidly evolving non-coding nuclear region *ITS* plays a valuable role in anchoring the universal standard coding regions *rbcL* and *matK* in a multigene tiered approach. This choice may vary among the groups under investigation. However, we found that the molecular phylogeny corresponded well with the latest morphological revisions in genus *Decalepis*^3,4^ and thus could be complemented with morphology to provide accurate identification of the species.

### Feasibility of analytical methods to provide clear discrimination of *Decalepis* species

An adequate bioinformatics resource to support the barcoding of life goes in parallel with finding a standard barcoding system for plants that goes beyond those relating to the use of a single marker (*COI*) for animal barcoding. Huge and overlapping datasets along with alignment difficulties of non-coding regions in plants necessitate the development of the best data analysis tools. Hitherto, different analytical methods have been employed for the assessment of species discrimination ability in plants group, with all of them showing certain pros and cons with the same dataset^46–50^. The two widely implemented approaches of distance- (TaxonDNA and NJ) and character-based methods (BLOG and PAUP), tested in this study both gave the highest correct identification rates. Our result seems to support the character-based approach as a highly workable and accurate method by producing a set of rules to characterize each species in terms of nucleotides at particular positions. For example, if position 548 = T and position 554 = A (as obtained in *rbcL* + *matK* + *ITS*), then the specimen is classified as *D. arayalpathra*. Also, there was 100% identification through BLOG for all the favourable loci, while TaxonDNA produced a few unidentified individuals (Table 3).

Among the three different modules (“BM”, “BCM”, and “All Species Barcodes”) implemented in TaxonDNA, the combination barcode *rbcL* + *matK* + *ITS*, correctly identified 15 species ~88.23% through “All Species Barcodes”. The “All Species Barcodes” criterion is known to be the strictest in providing correct identification as it requires query sequence matches to be above the proposed threshold^51^. The correct identification was 100% either through the BM, BCM and BLOG-based analysis among the *Decalepis* species. In order to assess the evolutionary process underlying the sequence datasets from the *Decalepis* group, MP analysis based on optimal criteria appeared to be the more reliable method, which produced several possible trees with correct topologies. The clustering algorithm used in the NJ method, which assumes K2P genetic distances between the sequences, might obscure ambiguities in data since it produces only one final tree^52^. The datasets used in the study, are ideal for these methods, as the sampled number of individuals per species was optimal, preventing the potential bias for all the methods. Overall, the results obtained in the study support the character-based approaches, BLOG and PAUP, as the method of choice in identifying the critically endangered species of genus *Decalepis*. The results of BLOG to characterize each species in terms of nucleotides at particular positions could be valuable in designing species-specific assays in CITES enforcement.

### Application of barcoding tools in conservation of *Decalepis*

Phylogenetic diversity defined by DNA barcode sequence data within and across the ecological communities at varying geographic scales can be an important measure in defining species boundaries and documenting new species, which in turn may result in the identification of targeted habitats for conservation^53^. Use of DNA barcoding to effectively discriminate threatened species to support the ongoing conservation measures has been successfully evaluated in many plant groups^42^. In the present study, we found that the combined barcode marker *rbcL* + *matK* + *ITS* supported a reticulated hypothesis of species in *Decalepis*, identifying it as a monophyletic group in accordance with previous studes^3,4^. As mentioned earlier, in the taxon sampling section of the manuscript, the individuals of *D. arayalpathra* were sampled from the population of regions Tirunelveli (Da1, Da2, Da3) and Kanyakumari districts (Da4, Da5), which showed a slightly higher level of genetic variation, resulting in a high priority for conservation concerns in our recent study of the population dynamics of *D. arayalpathra*^16^. Through DNA barcoding, it was interesting to conclude that the combined barcode marker *rbcL* + *matK* + *ITS* represented the genuine identity of the populations by clustering the individuals with 96% node support respective to their geographic range (Fig. 4). Da3 was placed as a polytomy at the base of the species cluster with an exception, which might be the result of a genetic pool with some migrants and admixed individuals^16^. The barcoding database generated through this study will help in gaining a more accurate assessment of the conservation status of *Decalepis* species based on molecular gene pools.

Species- or genus-specific single nucleotide polymorphisms (SNPs) based on chloroplast DNA are well suited for molecular marker development and have been shown to be an ideal source of genetic information that could be useful for species discrimination^54^. Genus- or species-specific assays enhance their applicability for direct use in CITES enforcement^55,56^. The 751 bp amplicons of the best single locus, *matK*, presented opportunities for species-specific sequence differentiation at different positions in the region between 225 bp to 630 bp. The sequences around the SNPs were checked for restriction sites. The most valuable SNP is the cytosine (C) located at position 230 of *D. hamiltonii* sequence which is exchanged by thymine (T) in the remaining species so that the suitable restriction enzyme *Bst*XI can be applied to discriminate between the species. The *D. arayalpathra* sequence also showed the presence of species-specific SNPs in the *matK* region at positions 90 and 587, but no suitable restriction sites were found to be available (although it may be possible to design species-specific PCR primers or HRM assays to detect them). Three species-specific SNPs were detected in the *D. salicifolia* sequence at positions 281, 437 and 627 with suitable restriction sites of *Mn1*I, *Hph*I and *Alw*I, respectively (dx.doi.org/10.5883/DS-CRCB). Thus, the specific sequence positions identified through the barcoding regions used in the present study can be used to design species-specific assays for testing the highly traded species of genus *Decalepis*.

## Conclusion

This study unequivocally demonstrates the efficiency of DNA barcoding for endemic species identification. The signature sequences of the proposed barcode *rbcL* + *matK* + *ITS* provided accurate signals in facilitating the molecular identity of *Decalepis* species in accordance with its latest taxonomic revision. The region clearly framed the entire set of sister species of *Decalepis* as a major cluster, with its potential substitute *H. indicus* in an out-group positioned as a nodal branch at the base of the tree. The character-based approach through PAUP and BLOG successfully distinguished 100% of investigated samples, rendering its accuracy and reliability as a method of choice in DNA barcoding studies. The species-specific assays derived from *matK* barcoding region sequences, further confirm its value in providing accurate species discrimination method. The inclusion of different conspecific populations is expected to gain insight into the conservation status of *Decalepis* species hotspots as well as emphasizing the practical application of DNA barcoding as a tool for the biodiversity conservation of endemic and threatened plant groups.

## Materials and Methods

### Taxon sampling and ethics statement

Species of genus *Decalepis* are highly endangered in its wild habitat. For sampling the plant material, an appropriate permission was granted by the Tamil Nadu Biodiversity Board (Letter Ref No. TNBB/52/2011 dated September 28, 2011, for a one year period from December 2011 to November 2012) and the Principal Chief Conservator of Forests and Chief Wildlife Warden, Tamil Nadu Forest Department (Letter Ref. No. WL5/ 23758/2011 dated December 5, 2011, for a one year period from December 2011 to November 2012) to study the reproductive biology, conservation issues, problems in germination, process of multiplication, DNA barcoding studies, etc. In our recent research publication, we performed population dynamics study on a total of sixty individuals corresponding to nine populations from different geographic regions to gain initial insight into their genetic diversity and population structure. It concluded that genetic diversity was remarkably low, but few populations from regions of Tirunelveli and Kanyakumari districts showed a slightly higher level of genetic variation resulting in high priority for conservation concerns^16^. Based on our findings, we considered the sampling of the same population in DNA barcoding studies for *D. arayalpathra*. Other plant species viz. *D. hamiltonii* and *D. salicifolia* were collected from their corresponding geographical hotspots. Plants of *H. indicus* were collected for out-group studies being the potential adulterant for *D. arayalpathra*.

A total of 15 individuals belonging to three different species of *Decalepis* were assembled from different geographical regions of Tamil Nadu, Kerala and Karnataka. Two individuals of *H. indicus* were collected from Tamil Nadu and Karnataka (Fig. 1). The samples were desiccated in silica gel and stored at −20 °C prior to DNA extraction. Vouchers specimens for each species sampled in this study were deposited at the herbarium maintained at Foundation for Revitalisation of Local Health Traditions (FRLHT), Bangalore, India and CSIR-Central Institute of Medicinal and Aromatic Plants (CIMAP), Lucknow, India, for future reference and the corresponding details are listed in Table 2.

### Molecular methods

Total genomic DNA was isolated from the reference samples using the cetyl trimethyl ammonium bromide (CTAB) protocol^57^. Isolated DNA was checked for its quality and quantity by electrophoresis on a 0.8% agarose gel and spectrophotometric analysis (NanoDrop, ND-1000, USA), respectively. The DNA was diluted to a final concentration of ~25–50 ng/µl for PCR amplification. Five candidate DNA barcode loci were amplified with the established primers, which included two coding cpDNA loci *rbcL* and *matK*; one non-coding cpDNA intergenic spacer loci, *psbA-trnH* and the nrDNA loci, *ITS* and *ITS2*. Details of primers and PCR conditions are listed in Table S1. PCR amplifications for each primer set were carried out in a 50 μl volume containing 1X Taq DNA polymerase buffer, 200 μM each dNTP (dATP:dTTP:dCTP:dGTP in 1:1:1:1 parts), 5–10 pmol of each primer (forward and reverse), 1 unit of Taq DNA polymerase and ~25–50 ng of template DNA. Successful amplicons were analysed by electrophoresis on 2% agarose gel. Subsequently, products of target molecular weight were purified with a Nucleospin PCR purification kit, using the manufacturer’s (MACHEREY-NAGEL – 07 / 2014, Rev.03) protocol and re-checked through electrophoresis on 2% agarose gel. The obtained product was subjected to Sanger’s di deoxy sequencing reactions, in forward and reverse directions using the BigDye Terminator v3.1 Cycle Sequencing Kit (Applied Biosystems, Foster City, CA) on an ABI 3130 XL genetic analyzer (Applied Biosystems).

### Databasing

Specimen data for each region were deposited in the Barcode of Life Data Systems (BOLD)^58^ (http://www.boldsystems.org) under the project CRCB – “DNA barcoding in *Decalepis*” (Table 2). All the data are accessible on BOLD under the dataset DS-CRCB (dx.doi.org/10.5883/DS-CRCB). The sequences were submitted to GenBank and are publicly accessible under the accession numbers listed in Table 2.

### Data analysis

The electropherograms obtained for each region were base-called using PHRED; sequences assembled and edited using Sequencher (Gene Codes Corporation, Ann Arbor, MI, USA). Finally, the sequences were blasted on NCBI BLAST under the programme BLASTN 2.2.1+^59,60^ and on to BOLD using Identification Request for checking their homology with other available sequences. All the barcode sequences were greater than 500 bp in length and free from contamination. The edited sequences were then aligned with Muscle 3.8.31 on the EMBLEBI website (http://www.ebi.ac.uk) under default parameters and adjusted manually in BioEdit v7.1.3.0^61^. The sequences were trimmed at both the ends to remove the primer sequences. All the variable sites were rechecked using the original trace files. Alignments can be obtained from the corresponding author upon reasonable request. Five candidate DNA barcode loci and their 26 possible combinations along with multi-gene tiered barcoding approach were evaluated based on the methods described ahead.

### Distance-based barcoding gap analyses

The distribution of within-species divergence to between-species divergence for the five candidate barcoding loci were studied using the ‘distance summary’ tool in BOLD. Using a barcode gap criterion, the intra-specific vs. nearest neighbour (NN) genetic distance was plotted to identify the barcoding gap. For the combinations of loci, the distribution of intra- and inte-rspecific variability was assessed by MEGA version6^62^ using the Kimura two-parameter distance model (K2P) of nucleotide substitution with pairwise deletion of missing sites^63^. Barcoding gaps for all the loci were recorded by plotting the inter- and intra-specific distances with frequency distribution in bin interval of 0.005; estimated using the “pairwise summary” in Species Identifier 1.7.7 program from the TaxonDNA software package^64^. Since the coalescent depths vary among species, substantial overlap between intra- and inter-specific distances might not compromise with the species identification success. Therefore, the local barcoding gap for each species was evaluated for all the combinations of barcodes, by plotting the distance to the nearest non-conspecific against the distance to the NN with a 1:1 slope^65^. The proportion of correct species identifications were annotated using “Best Match” (BM), “Best Close Match” (BCM), and “All Species Barcodes” functions embedded in TaxonDNA. The tool examines all the sequences present in the aligned dataset and compares each successive sequence with all the other sequences to determine the closest match. The BM module then classifies the sequences as correct and incorrect based on the indicated pair from the similar species or different species, respectively. While the various equally best matches from different species are referred to as ambiguous, the BCM module works on the intra-species variability criterion and is considered to be the more rigorous method in TaxonDNA.

### Character-based approach through BLOG

Barcoding with LOGic (BLOG), is a character-based machine learning approach with program BLOG2.0 to classify specimen’s sequences to species, using a set of classification rules in terms of DNA barcode locations of key diagnostic nucleotides^66,67^. It formulates the classification rules based on the supplied training dataset and then applies the same to both the training set and the test set to estimate the identification success. The different barcode datasets used in this study were subjected to 90% slicing within species-level with a maximum of 500 iterations (GRASPITER = 500) and a maximum time of 5 minutes for analysis (GRASPSECS = 300). The logic formula with lowest false positive rate against the reference dataset was taken as identification basis.

### Phylogenetic trees using distance- and character-based methods

To delimit the species into discrete clades or monophyletic groups, phylogenetic analysis was carried out on the studied datasets. The evolutionary process of the sequence data was assessed based on distance-based and character-based methods. Neighbour-joining with minimum evolution (NJ) clustering algorithm was used to calculate the evolutionary distance between sequences. The NJ trees were constructed in PAUP 4.0^68^ based on K2P distances as genetic measure and setting negative branch lengths to zero.

Among the character-based approaches, maximum parsimony (MP) method was used to determine the most probable evolutionary event history between sequences. MP analysis was performed in PAUP 4.0 with the HKY-gamma substitution model to account for rate variation among sites. An initial heuristic search was made with 1000 replicates and branch swapping was performed by tree-bisection-reconnection (TBR). A maximum of 10 trees were held at each step with random stepwise addition for the starting tree in each replicate. The trees found in the first round were subjected to a second search by TBR swapping holding up to 15000 trees and swapping to completion. The reliability of the node was assessed by a bootstrap test with 1000 pseudo-replicates^69–71^. *H. indicus* was used as outgroup for all the methods.

### Data Accessibility

GenBank (NCBI) [http://www.ncbi.nlm.nih.gov/genbank] Accession numbers for nucleotide sequences are listed in Table 2. Sequence alignments have been archived in BOLD [http://www.boldsystems.org] project DS-CRCB (dx.doi.org/10.5883/DS-CRCB).

## Electronic supplementary material

Character-based DNA barcoding for authentication and conservation of IUCN Red listed threatened species of genus Decalepis (Apocynaceae)
