# Author Correction: Character-based DNA barcoding for authentication and conservation of IUCN Red listed threatened species of genus *Decalepis* (Apocynaceae)

**DOI:** 10.1038/s41598-020-74406-0

**Published:** 2020-10-16

**Authors:** Priyanka Mishra, Amit Kumar, Gokul Sivaraman, Ashutosh K. Shukla, Ravikumar Kaliamoorthy, Adrian Slater, Sundaresan Velusamy

**Affiliations:** 1 Plant Biology and Systematics, CSIR - Central Institute of Medicinal and Aromatic Plants, Research Center, Allalsandra, GKVK Post, Bengaluru, Karnataka 560065 India; 2 Biotechnology Division, CSIR - Central Institute of Medicinal and Aromatic Plants, P.O. CIMAP, Lucknow, Uttar Pradesh 226015 India; 3 School of Conservation, TransDisciplinary University, 74/2, Jarakabande Kaval, Post Attur, Via Yelahanka, Bangalore, Karnataka 560064 India; 4 Biomolecular Technology Group, Faculty of Health and Life Sciences, De Montfort University, Leicester, LE1 9BH UK

Correction to: *Scientific Reports* 10.1038/s41598-017-14887-8, published online 02 November 2017

This Article contains errors in the GenBank accession numbers reported for *Decalepis salicifolia* and *Hemidesmus indicus* in Table 2.

The ‘*ITS*’ GenBank accession number change for Sampling Location, ‘Kathadimudi Peak; Coimbatore’, BOLD database sample Id ‘CRCBDs2’,

“KT338799”

should read:

“MT878150”

The ‘*ITS*’ GenBank accession number change for Sampling Location, ‘Vattakandal Shola; Kodaikanal’,

“KT338798”

should read:

“MT878151”

The ‘*ITS2*’ GenBank accession number change for Sampling Location, ‘Valasamalai; Tiruvannamalai’,

“KT362305”

should read:

“MT892812”

The ‘*ITS2*’ GenBank accession number change for Sampling Location, ‘Hosur; Krishnagiri’,

“KT362306”

should read:

“KT362305”

