# Supplementary material for: Character-based DNA barcoding for authentication and conservation of IUCN Red listed threatened species of genus Decalepis (Apocynaceae)
Source: Sci Rep. 2017 Nov 2;7:14910. doi: 10.1038/s41598-017-14887-8 (PMC5668324; doi:10.1038/s41598-017-14887-8)
Supplement: Supplementary file 1 — Character-based DNA barcoding for authentication and conservation of IUCN Red listed threatened species of genus Decalepis (Apocynaceae) [file 41598_2017_14887_MOESM1_ESM.pdf]

# Character-based DNA barcoding for authentication and conservation of IUCN Red listed threatened species of genus *Decalepis* (Apocynaceae)

**Priyanka Mishra[1], Amit Kumar[1], Sivaraman Gokul[1], Ashutosh K. Shukla[2], Kaliamoorthy Ravikumar[3], Adrian Slater[4] & Velusamy Sundaresan[1,*]**

[1] *Plant Biology and Systematics, CSIR - Central Institute of Medicinal and Aromatic Plants, Research Center, Allalsandra, GKVK Post, Bengaluru - 560065, Karnataka, India*

[2] *Biotechnology Division, CSIR - Central Institute of Medicinal and Aromatic Plants, P.O. CIMAP, Lucknow - 226015, Uttar Pradesh, India*

[3] *School of Conservation, TransDisciplinary University, 74/2, Jarakabande Kaval, Post Attur, Via Yelahanka, Bangalore - 560064, Karnataka, India*

[4] *Biomolecular Technology Group, Faculty of Health and Life Sciences, De Montfort University, Leicester, LE1 9BH, UK*

[*] Correspondence: Dr. V. Sundaresan
Senior Scientist
Plant Biology and Systematics
CSIR - Central Institute of Medicinal and Aromatic Plants, Research Center, Bengaluru - 560065, Karnataka, India
Tel: +91 – 80 – 22746956; Fax: +91 – 80 – 28564707
vsundaresan@cimap.res.in

CIMAP Communication Number: CIMAP/PUB/2016/85

## Supplementary information

**Figure S1** Morphological view of: (a - b) *Decalepis arayalpathra* fresh roots in wild (c) Raw drug samples of *D. arayalpathra* roots in markets (d) *D. hamiltonii* fresh roots in wild (e) *D. hamiltonii* dried roots in wild (f) Raw drug samples of *D. hamiltonii* roots in markets (g) *D. salicifolia* fresh roots in wild (h) *Hemidesmus indicus* fresh roots in wild (i) Raw drug samples of *H. indicus* roots in markets (j) Raw drug samples of *H. indicus* stems in markets.

**Figure S2** Relative distribution of intra-specific and inter-specific distances for all the single and combination of favourable barcodes in *Decalepis*; x-axes relate to K2P distances arranged in intervals, and the y-axes correspond to the percentage of occurrences.

**Figure S3** Maximum parsimony trees constructed using candidate barcode markers showing species resolution rates in terms of single locus. Bootstrap support values below 70% are not shown.

**Table S1** Primers used for amplification and sequencing for DNA barcoding of *Decalepis* species.

**Table S2** Mean and maximum intra-specific and nearest neighbor (NN) distance for all the species using candidate barcodes.

**Table S3** Genetic distance (%) measured using Kimura 2-parameter model for the candidate barcode loci and their combinations.

**Table S4** Identification success rates based on analysis of the 'Best Match', 'Best Close Match' and 'All Species Barcodes' function of TaxonDNA software for each DNA barcoding marker and its combinations.

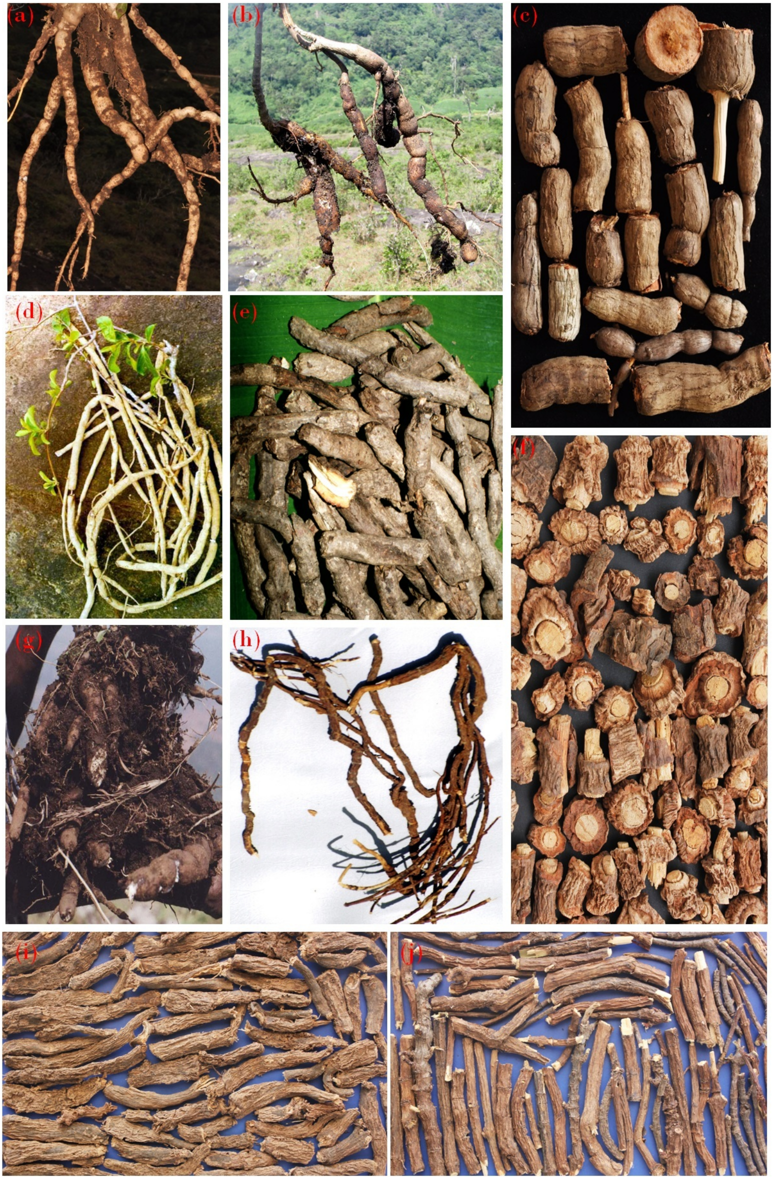
(a)
(b)
(c)
(d)
(e)
(f)
(g)
(h)
(i)
(j)

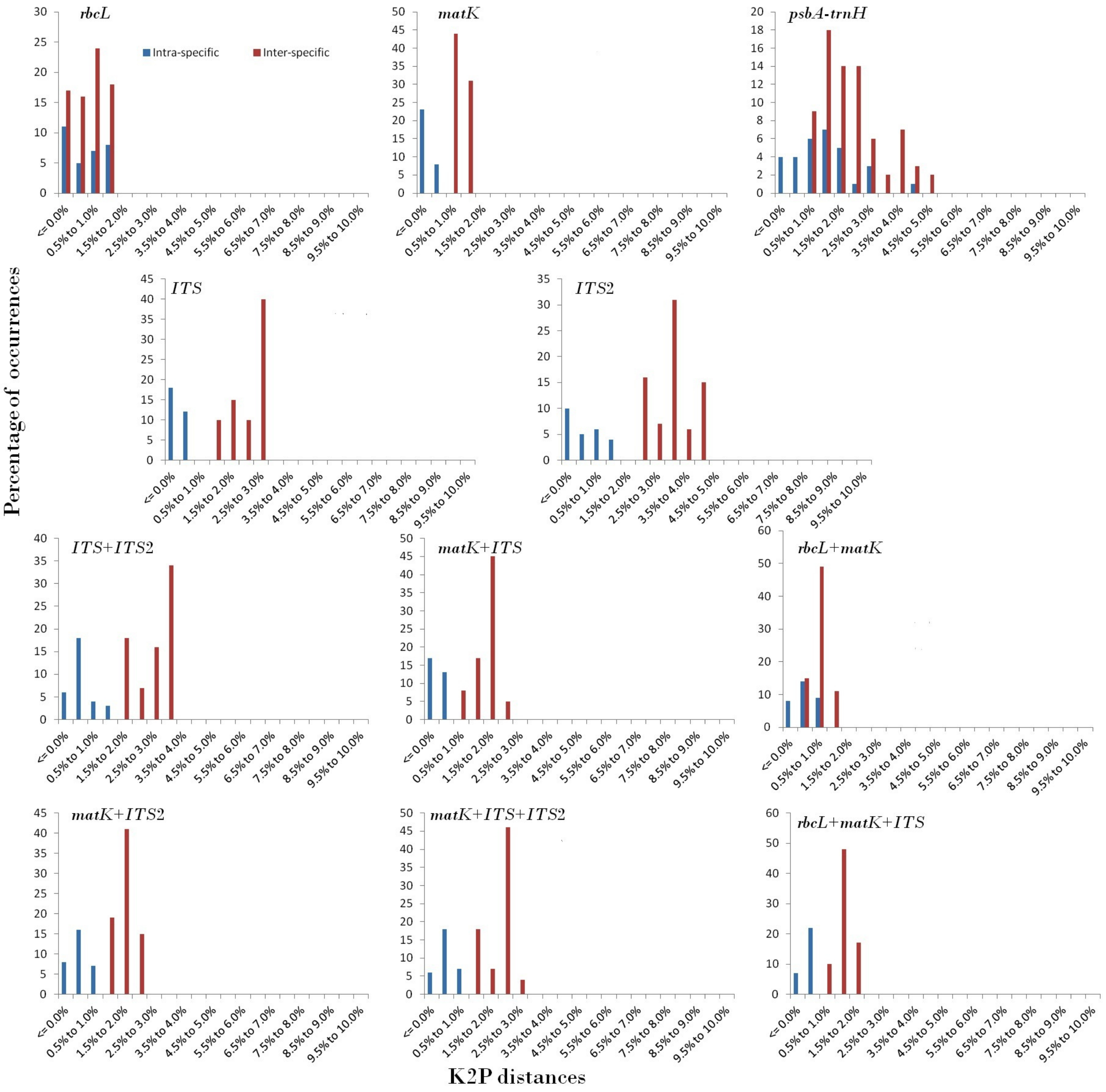

rbcL
Intra-specific
Inter-specific
matK
psbA-trnH
ITS
ITS2
ITS+ITS2
matK+ITS
rbcL+matK
matK+ITS2
matK+ITS+ITS2
rbcL+matK+ITS
Percentage of occurrences
K2P distances
<=0.0%
0.5% to 1.0%
1.5% to 2.0%
2.5% to 3.0%
3.5% to 4.0%
4.5% to 5.0%
5.5% to 6.0%
6.5% to 7.0%
7.5% to 8.0%
8.5% to 9.0%
9.5% to 10.0%

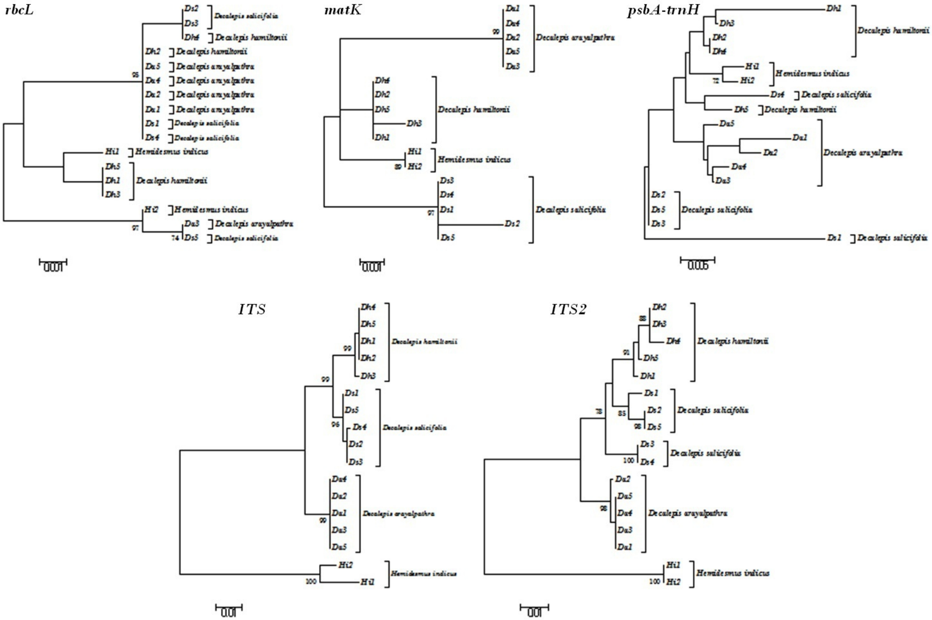
rbcL
Ds2
Ds3
Decalepis salicifolia
Dh4
Decalepis hamiltonii
Dh2
Decalepis hamiltonii
Da5
Decalepis arayalpathra
Da4
Decalepis arayalpathra
Da2
Decalepis arayalpathra
Da1
Decalepis arayalpathra
Ds1
Decalepis salicifolia
Ds4
Decalepis salicifolia
95
Hi1
Hemidesmus indicus
Dh5
Dh1
Dh3
Decalepis hamiltonii
Hi2
Hemidesmus indicus
Da3
Decalepis arayalpathra
97
74
Ds5
Decalepis salicifolia
0.001
matK
Da1
Da4
Da2
Da5
Da3
99
Decalepis arayalpathra
Dh4
Dh2
Dh5
Dh3
Dh1
Decalepis hamiltonii
Hi1
Hi2
89
Hemidesmus indicus
Ds3
Ds4
Ds1
Ds2
Ds5
97
Decalepis salicifolia
0.001
psbA-trnH
Dh1
Dh3
Dh2
Dh4
Decalepis hamiltonii
Hi1
Hi2
72
Hemidesmus indicus
Ds4
Decalepis salicifolia
Dh5
Decalepis hamiltonii
Da5
Da1
Da2
Da4
Da3
Decalepis arayalpathra
Ds2
Ds5
Ds3
Decalepis salicifolia
Ds1
Decalepis salicifolia
0.005
ITS
Dh4
Dh5
Dh1
Dh2
Dh3
99
99
Decalepis hamiltonii
Ds1
Ds5
Ds4
Ds2
Ds3
96
Decalepis salicifolia
Da4
Da2
Da1
Da3
Da5
99
Decalepis arayalpathra
Hi2
Hi1
100
Hemidesmus indicus
0.01
ITS2
Dh2
Dh3
88
Dh4
Dh5
Dh1
91
Decalepis hamiltonii
Ds1
Ds2
Ds5
85
98
78
Decalepis salicifolia
Ds3
Ds4
100
Decalepis salicifolia
Da2
Da5
Da4
Da3
Da1
98
Decalepis arayalpathra
Hi1
Hi2
100
Hemidesmus indicus
0.01

**Table S1. Primers used for amplification and sequencing for DNA barcoding of *Decalepis* species.**

| Region | Primer name | Sequence (5´-3´) | Thermal cycling conditions | Mean size | Reference |
|---|---|---|---|---|---|
| *rbcL* | 1F | 5'-ATGTCACCACAAACAGAAAC-3' | 95°C 2 min; [35 cycles: 94°C 1 min; 55°C 30 s; 72°C 1 min]; 72°C 7 min | 715 bp | 25 |
| | 724R | 5'-TCGCATGTACCTGCAGTAGC-3' | | | |
| *matK* | 390F | 5'-CGATCTATTCATTCAATATTTC-3' | 95°C 2 min; [30 cycles: 94°C 1 min; 48°C 30 s; 72°C 1 min]; 72°C 7 min | 856 bp | 70 |
| | 1326R | 5'-TCTAGCACACGAAAGTCGAAGT-3' | | | |
| *psbA-trnH* | *psbA*F | 5'-GTTATGCATGAACGTAATGCTC-3 | 94°C 5 min; [35 cycles: 94°C 1 min; 55°C 30 s; 72°C 1.5 min]; 72°C 7 min | 400 bp | 71 |
| | *trnH*R | 5'-CGCGCATGGTGGATTCACAATCC-3' | | | |
| *ITS* | *ITS5a* | 5'-CCTTATCATTTAGAGGAAGGAG-3' | 94°C 5 min; [30 cycles: 94°C 1 min; 50°C 1 min; 72°C 1.5 min]; 72°C 7 min | 704 bp | 25 |
| | *ITS4* | 5'- ATGCGATACTTGGTGTGAAT -3' | | | |
| *ITS*2 | *S2F* | 5'-TCCTCCGCTTATTGATATGC-3' | 94°C 5 min; [40 cycles: 94°C 30 sec; 56°C 30 sec; 72°C 45 sec]; 72°C 10 min | 450 bp | 53 |
| | *S3R* | 5'- GACGCTTCTCCAGACTACAAT-3' | | | |

**Table S2. Mean and maximum intra-specific and nearest neighbor (NN) distance for all the species using candidate barcodes.**

| Barcode | Species | Mean intra-specific distance | Max intra-specific distance | Nearest Neighbour | Distance to NN |
|---|---|---|---|---|---|
| *rbcL* | *Decalepis arayalpathra* | 0.48 | 1.19 | *D. salicifolia* | 0 |
| | *Decalepis hamiltonii* | 0.51 | 0.89 | *D. salicifolia* | 0 |
| | *Decalepis salicifolia* | 0.57 | 1.34 | *D. arayalpathra* | 0 |
| | *Hemidesmus indicus* | 0.89 | 0.89 | *D. salicifolia* | 0.15 |
| *matK* | *Decalepis arayalpathra* | 0 | 0 | *D. hamiltonii* | 0.8 |
| | *Decalepis hamiltonii* | 0.05 | 0.13 | *H. indicus* | 0.4 |
| | *Decalepis salicifolia* | 0.11 | 0.27 | *D. hamiltonii* | 0.67 |
| | *Hemidesmus indicus* | 0 | 0 | *D. hamiltonii* | 0.4 |
| *psbA-trnH* | *Decalepis arayalpathra* | 0.97 | 1.62 | *D. salicifolia* | 0.8 |
| | *Decalepis hamiltonii* | 1..02 | 1.16 | *D. salicifolia* | 0.8 |
| | *Decalepis salicifolia* | 1.63 | 4.11 | *D. hamiltonii* | 0.8 |
| | *Hemidesmus indicus* | 0.53 | 0.53 | *D. hamiltonii* | 0.8 |
| *ITS* | *Decalepis arayalpathra* | 0 | 0 | *D. salicifolia* | 2.47 |
| | *Decalepis hamiltonii* | 0.12 | 0.3 | *D. salicifolia* | 1.37 |
| | *Decalepis salicifolia* | 0.15 | 0.3 | *D. hamiltonii* | 1.37 |
| | *Hemidesmus indicus* | 2.15 | 2.15 | *D. arayalpathra* | 11.67 |
| *ITS2* | *Decalepis arayalpathra* | 0.1 | 0.25 | *D. hamiltonii* | 3.08 |
| | *Decalepis hamiltonii* | 0.6 | 1.27 | *D. salicifolia* | 2.03 |
| | *Decalepis salicifolia* | 1.57 | 2.29 | *D. hamiltonii* | 2.03 |
| | *Hemidesmus indicus* | 0 | 0 | *D. arayalpathra* | 10.69 |

**Table S3. Genetic distance (%) measured using Kimura 2-parameter model for the candidate barcode loci and their combinations.**

| Barcode locus/loci | Range of Intra-specific distances (±S.D.) | Range of Inter-specific distances (±S.D.) |
|---|---|---|
| *rbcL* | 0.004 (±0.001) – 0.008 (±0.003) | 0.004 (±0.001) – 0.009 (±0.003) |
| *matK* | 0.000 (±0.000) – 0.001 (±0.000) | 0.004 (±0.002) – 0.013 (±0.004) |
| *psbA-trnH* | 0.005 (±0.003) – 0.016 (±0.004) | 0.015 (±0.005) – 0.021 (±0.006) |
| *ITS* | 0.000 (±0.000) – 0.215 (±0.005) | 0.015 (±0.004) – 0.132 (±0.015) |
| *ITS2* | 0.000 (±0.000) – 0.015 (±0.004) | 0.025 (±0.006) – 0.122 (±0.019) |
| *rbcL+ matK* | 0.002 (±0.000) – 0.004 (±0.001) | 0.006 (±0.002) – 0.009 (±0.002) |
| *rbcL+ psbA-trnH* | 0.006 (±0.001) – 0.009 (±0.001) | 0.010 (±0.002) – 0.013 (±0.003) |
| *rbcL+ITS* | 0.002 (±0.000) – 0.015 (±0.003) | 0.011 (±0.002) – 0.066 (±0.007) |
| *rbcL+ITS2* | 0.003 (±0.001) – 0.009 (±0.002) | 0.013 (±0.003) – 0.047 (±0.006) |
| *matK+ psbA-trnH* | 0.001 (±0.001) – 0.006 (±0.001) | 0.008 (±0.002) – 0.015 (±0.003) |
| *matK+ITS* | 0.000 (±0.000) – 0.009 (±0.002) | 0.011 (±0.003) – 0.061 (±0.006) |
| *matK+ITS2* | 0.000 (±0.000) – 0.006 (±0.001) | 0.013 (±0.003) – 0.043 (±0.006) |
| *psbA-trnH+ITS* | 0.003 (±0.001) – 0.015 (±0.004) | 0.017 (±0.003) – 0.087 (±0.009) |
| *psbA-trnH+ITS2* | 0.002 (±0.001) – 0.015 (±0.003) | 0.023 (±0.004) – 0.067 (±0.009) |
| *ITS+ITS2* | 0.000 (±0.000) – 0.013 (±0.003) | 0.019 (±0.004) – 0.128 (±0.011) |
| *rbcL+ matK+ psbA-trnH* | 0.003 (±0.000) – 0.005 (0.001) | 0.008 (±0.002) – 0.011 (±0.002) |
| *rbcL+ matK+ITS* | 0.001 (±0.000) – 0.009 (±0.002) | 0.010 (±0.002) – 0.044 (±0.004) |
| *rbcL+ matK+ITS2* | 0.001 (±0.000) – 0.005 (±0.001) | 0.011 (±0.002) – 0.030 (±0.004) |
| *rbcL+ psbA-trnH+ITS* | 0.003 (±0.000) – 0.012 (±0.002) | 0.013 (±0.002) – 0.054 (±0.005) |
| *rbcL+ psbA-trnH+ITS2* | 0.004 (±0.001) – 0.011 (±0.001) | 0.015 (±0.003) – 0.039 (±0.005) |
| *rbcL+ITS+ITS2* | 0.002 (±0.000) – 0.011 (±0.002) | 0.014 (±0.002) – 0.078 (±0.006) |
| *matK+ psbA-trnH+ITS* | 0.002 (±0.000) – 0.009 (±0.002) | 0.013 (±0.002) – 0.052 (±0.005) |
| *matK+ psbA-trnH+ITS2* | 0.001 (±0.000) – 0.008 (±0.001) | 0.015 (±0.002) – 0.038 (±0.005) |
| *matK+ITS+ITS2* | 0.000 (±0.000) – 0.007 (±0.001) | 0.014 (±0.003) – 0.074 (±0.007) |
| *psbA-trnH+ITS+ITS2* | 0.002 (±0.000) – 0.011 (±0.003) | 0.019 (±0.003) – 0.096 (±0.008) |
| *rbcL+ matK+ psbA-trnH+ITS* | 0.002 (±0.000) – 0.008 (±0.002) | 0.011 (±0.002) – 0.040 (±0.004) |
| *rbcL+ matK+ psbA-trnH+ITS2* | 0.003 (±0.000) – 0.007 (±0.001) | 0.013 (±0.002) – 0.029 (±0.003) |
| *rbcL+ psbA-trnH+ITS+ITS2* | 0.003 (±0.000) – 0.010 (±0.002) | 0.015 (±0.002) – 0.066 (±0.006) |
| *rbcL+ matK+ITS+ITS2* | 0.001 (±0.000) – 0.008 (±0.001) | 0.012 (±0.002) – 0.055 (±0.004) |
| *matK+ psbA-trnH+ITS+ITS2* | 0.001 (±0.000) – 0.007 (±0.001) | 0.015 (±0.002) – 0.063 (±0.005) |
| *rbcL+ matK+ psbA-trnH+ITS+ITS2* | 0.002 (±0.000) – 0.007 (±0.001) | 0.013 (±0.002) – 0.050 (±0.004) |

**Table S4. Identification success rates based on analysis of the 'Best match', 'Best close match' and 'All species barcodes' function of TaxonDNA software for each DNA barcoding marker and its combinations.**

| Barcode locus/loci | Threshold (%) | Best match (%) | | | Best close match (%) | | | | All species barcode (%) |
|---|---|---|---|---|---|---|---|---|---|
| | | Successfully identified (%) | Ambiguous (%) | Misidentified (%) | Successfully identified (%) | Ambiguous (%) | Misidentified (%) | No match (%) | |
| *rbcL* | 1.34 | 3 (17.64) | 10 (58.52) | 4 (23.52) | 3 (17.64) | 10 (58.52) | 4 (23.52) | 0 (0.00) | 0.00 |
| *matK* | 0.25 | 17 (100) | 0 (0.00) | 0 (0.00) | 15 (88.23) | 0 (0.00) | 0 (0.00) | 2 (11.76) | 76.47 |
| *psbA-trnH* | 4.09 | 16 (94.11) | 0 (0.00) | 1(5.88) | 16 (94.11) | 0 (0.00) | 1 (5.88) | 0 (0.00) | 17.64 |
| *ITS* | 2.15 | 17 (100) | 0 (0.00) | 0 (0.00) | 15 (88.23) | 0 (0.00) | 0 (0.00) | 2 (11.76) | 88.23 |
| *ITS2* | 2.27 | 17 (100) | 0 (0.00) | 0 (0.00) | 17 (100) | 0 (0.00) | 0 (0.00) | 0 (0.00) | 58.82 |
| *rbcL+ matK* | 0.76 | 14 (82.35) | 1 (5.88) | 2 (11.76) | 14 (82.35) | 1 (5.88) | 2 (11.76) | 0 (0.00) | 5.88 |
| *rbcL+ psbA-trnH* | 1.73 | 13 (76.47) | 0 (0.00) | 4 (23.52) | 13 (76.47) | 0 (0.00) | 4 (23.52) | 0 (0.00) | 11.76 |
| *rbcL+ITS* | 1.50 | 17 (100) | 0 (0.00) | 0 (0.00) | 15 (88.23) | 0 (0.00) | 0 (0.00) | 2 (11.76) | 88.23 |
| *rbcL+ITS2* | 1.69 | 17 (100) | 0 (0.00) | 0 (0.00) | 17 (100) | 0 (0.00) | 0 (0.00) | 0 (0.00) | 64.7 |
| *matK+ psbA-trnH* | 1.34 | 17 (100) | 0 (0.00) | 0 (0.00) | 13 (76.47) | 0 (0.00) | 4 (23.52) | 0 (0.00) | 64.7 |
| *matK+ITS* | 0.99 | 17 (100) | 0 (0.00) | 0 (0.00) | 15 (88.23) | 0 (0.00) | 0 (0.00) | 2 (11.76) | 88.23 |
| *matK+ITS2* | 0.95 | 17 (100) | 0 (0.00) | 0 (0.00) | 17 (100) | 0 (0.00) | 0 (0.00) | 0 (0.00) | 88.23 |
| *psbA-trnH+ITS* | 1.64 | 14 (82.35) | 1 (5.88) | 2 (11.76) | 14 (82.35) | 1 (5.88) | 2 (11.76) | 0 (0.00) | 82.35 |
| *psbA-trnH+ITS2* | 3.16 | 13 (76.47) | 0 (0.00) | 4 (23.52) | 13 (76.47) | 0 (0.00) | 4 (23.52) | 0 (0.00) | 58.82 |
| *ITS+ITS2* | 1.33 | 17 (100) | 0 (0.00) | 0(0.00) | 15 (88.23) | 0(0.00) | 0 (0.00) | 2 (11.76) | 88.23 |
| *rbcL+ matK+ psbA-trnH* | 1.00 | 17 (100) | 0 (0.00) | 0 (0.00) | 15 (88.23) | 0 (0.00) | 0 (0.00) | 2 (11.76) | 47.05 |
| *rbcL+ matK+ITS* | 0.95 | 17 (100) | 0 (0.00) | 0 (0.00) | 17 (100) | 0 (0.00) | 0 (0.00) | 0 (0.00) | 88.23 |
| *rbcL+ matK+ITS2* | 0.99 | 17 (100) | 0 (0.00) | 0 (0.00) | 17 (100) | 0 (0.00) | 0 (0.00) | 0 (0.00) | 76.47 |
| *rbcL+ psbA-trnH+ITS* | 1.28 | 17 (100) | 0 (0.00) | 0 (0.00) | 15 (88.23) | 0 (0.00) | 0 (0.00) | 2 (11.76) | 88.23 |
| *rbcL+ psbA-trnH+ITS2* | 1.66 | 17 (100) | 0 (0.00) | 0 (0.00) | 17 (100) | 0 (0.00) | 0 (0.00) | 0 (0.00) | 47.05 |
| *rbcL+ITS+ITS2* | 1.15 | 17 (100) | 0 (0.00) | 0 (0.00) | 15 (88.23) | 0 (0.00) | 0 (0.00) | 2 (11.76) | 88.23 |
| *matK+ psbA-trnH+ITS* | 0.94 | 17 (100) | 0 (0.00) | 0 (0.00) | 17 (100) | 0 (0.00) | 0 (0.00) | 0 (0.00) | 88.23 |
| *matK+ psbA-trnH+ITS2* | 1.58 | 17 (100) | 0 (0.00) | 0 (0.00) | 17 (100) | 0 (0.00) | 0 (0.00) | 0 (0.00) | 82.35 |
| *matK+ITS+ITS2* | 0.76 | 17 (100) | 0 (0.00) | 0 (0.00) | 15 (88.23) | 0 (0.00) | 0 (0.00) | 2 (11.76) | 88.23 |
| *psbA-trnH+ITS+ITS2* | 1.83 | 17 (100) | 0 (0.00) | 0 (0.00) | 17 (100) | 0(0.00) | 0(0.00) | 0(0.00) | 82.35 |
| *rbcL+ matK+ psbA-trnH+ITS* | 0.88 | 17 (100) | 0 (0.00) | 0 (0.00) | 15 (88.23) | 0(0.00) | 0(0.00) | 2(11.76) | 88.23 |

| | | | | | | | | | |
|---|---|---|---|---|---|---|---|---|---|
| *rbcL+ matK+ psbA-trnH+ITS2* | 1.09 | 17 (100) | 0 (0.00) | 0 (0.00) | 17 (100) | 0 (0.00) | 0 (0.00) | 0 (0.00) | 88.23 |
| *rbcL+ psbA-trnH+ITS+ITS2* | 1.24 | 17 (100) | 0 (0.00) | 0 (0.00) | 17 (100) | 0 (0.00) | 0 (0.00) | 0 (0.00) | 88.23 |
| *rbcL+ matK+ITS+ITS2* | 0.80 | 17 (100) | 0 (0.00) | 0 (0.00) | 15 (88.23) | 0 (0.00) | 0 (0.00) | 2 (11.76) | 88.23 |
| *matK+ psbA-trnH+ITS+ITS2* | 1.19 | 17 (100) | 0 (0.00) | 0 (0.00) | 17 (100) | 0 (0.00) | 0 (0.00) | 0 (0.00) | 88.23 |
| *rbcL+ matK+ psbA-trnH+ITS+ITS2* | 0.91 | 17 (100) | 0 (0.00) | 0 (0.00) | 17 (100) | 0 (0.00) | 0 (0.00) | 0 (0.00) | 88.23 |

Identification analysis for each barcoding marker and its combinations was performed at their respective threshold calculated from pairwise distances.
